# Inflammation and Invasion in Oral Squamous Cell Carcinoma Cells Exposed to Electronic Cigarette Vapor Extract

**DOI:** 10.3389/fonc.2022.917862

**Published:** 2022-07-22

**Authors:** Hannah P. Robin, Courtney N. Trudeau, Adam J. Robbins, Emily J. Chung, Erum Rahman, Olivia L. Gangmark Strickland, Scott Jordan, Frank W. Licari, Duane R. Winden, Paul R. Reynolds, Juan A. Arroyo

**Affiliations:** ^1^ College of Dental Medicine, Roseman University of Health Sciences, South Jordan, UT, United States; ^2^ Lung and Placenta Laboratory, Department of Cell Biology and Physiology, Brigham Young University, Provo, UT, United States

**Keywords:** OSCC, eCIG, inflammation, invasion, gingiva

## Abstract

Electronic cigarettes (eCig) represent a new avenue of tobacco exposure that involves heating oil-based liquids and the delivery of aerosolized flavors with or without nicotine, yet little is known about their overall health impact. The oral cavity is an anatomic gateway for exposure that can be compromised by activating myriad of signaling networks. Oral squamous cell carcinoma (OSSC) is a common malignancy affecting 30,000 people in the United States each year. Our objective was to determine the impact of eCig and nicotine on gingival OSSC invasion and their secretion of pro-inflammatory molecules. Gingiva-derived Ca9-22 cells and tongue-derived Cal27 cells were exposed to eCig vapor extract (EVE) generated from Red Hot or Green Apple (Apple) flavored eCig solution +/- nicotine for 6 hours. Isolation of protein lysates and collection conditioned media was done after treatment. Real-time cellular invasion was assessed using a RTCA DP instrument. Protein expression was determined using western blot. Compared to controls, we observed: elevated NF-*k*B, TNF-α, ERK, JNK, MMP-13 and cell invasion by Ca9-22 treated with Apple EVE; increased TNF-α and JNK by Ca9-22 treated with Red Hot EVE; and increased TNF-α and JNK by Cal27 cells treated with both Apple and Red Hot EVE. We conclude that eCig flavoring and nicotine orchestrated differential cell invasion and inflammatory effects. This study provides an important initial step in dissecting mechanisms of cancerous invasion and molecular avenues employed by OSCC.

## Introduction

Head and neck cancer is the 6th most common cancer worldwide, causing nearly 380,000 deaths each year (1). Oral squamous cell carcinoma (OSCC) encompasses about 90% of oral cavity cancers and affects approximately 30,000 people every year (2–5). OSCC is characterized by frequent early metastatic migration to distant organs; therefore, understanding the development and migratory aspects of this type of cancer is critical in potential treatment modalities (4). Furthermore, OSSC has shown a high recurrence rate (up to 50%) leading to OSCC progression and patients eventually die of tumor recurrence and subsequent metastasis (5, 6). OSCC is closely related to environmental factors including tobacco consumption, alcohol habits, nutrition, and viral infections (5, 7, 8). Of these factors, cigarette smoke is associated with is 7–10 times higher OSCC development when compared to non-smokers (5, 8). Despite concerted social efforts to reduce smoking prevalence, current trends suggest that smoking numbers will continue to increase. Cigarette smoke is a pervasive inhaled toxin; almost half of the U.S. population is regularly exposed to first or secondhand smoke (SHS) and approximately 20% of young children live with someone who smokes in the home (9–11). Recently, the use of alternative tobacco products such as electronic cigarettes (eCigs) as a “healthier” alternate to traditional tobacco smoking has become popular. The use of eCigs among adolescents is the most widespread alternative tobacco behavior and its use among adults has increased up to 8.2% of the general population (12).

Although these nicotine delivery methods are proposed to be healthier, recent research has shown adverse health effects associated with eCig use including bronchitis, mouth/throat irritation, headaches, nausea, airway obstruction, bronchospasm, inflammation, and cardiovascular effects (elevated heart rate, blood pressure, and vessel stiffness) (6, 11, 13–17). Research from our laboratory revealed increased inflammation and cell invasion by OSCC cells treated with eCig liquid. In the current report, we show that eCig flavoring and nicotine orchestrate differential regulation of OSCC cell invasion and inflammatory effects. Despite abundant data highlighting exposure to cigarette smoke and downstream adverse effects on OSSC severity, reports detailing the consequences of eCig vapor are less common. To better understand eCig vapor effects, we examined the effects of eCig vapor extract (EVE) of two common eCig flavors, sweet apple and cinnamaldehyde, with or without nicotine, on OSCC signaling, invasiveness, and inflammatory outcome.

## Materials and methods

### Cell Culture and eCig Vapor Extract (EVE)

Ca9-22 human oral squamous carcinoma cells and Cal27 human tongue squamous carcinoma cells were used in these experiments (both from ATCC, Manassas, VA). Cells were maintained in RPMI medium plus 10% fetal bovine serum (FBS) (Invitrogen, Carlsbad, CA, USA). EVE was generated as follows: an eCig module was connected, by the mouthpiece, to a vacuum pump while pressing the button on the eCig module for 3 seconds. The vacuum pump drew vapor from the mouthpiece of the eCig module through the tip of a pipette submerged in a tube containing 10 ml of serum free medium. This process was repeated, involving 3 second puffs, followed by 20 second rest, for a total of 20 puffs. The conditioned medium was identified as 100% EVE solution. This procedure was performed for each flavor (Red Hot; 8Ohm1, Inc., Ogden, UT, or Reds Apple Juice; Green Apple, Daze Mfg., Los Angeles) in the presence or absence of 6 mg of nicotine and compared to untreated cells (Control).

### Cell Treatments

At approximately 80% cell confluency, the two cell lines were incubated for 6 hours in medium alone (control), or medium supplemented with a dose curve of Red Hot or Green Apple (Apple) eCig vapor extract (EVE, 5-50%) in the presence or absence of 6mg nicotine. These flavors are amongst the more popular varieties and a dose curve identified a concentration of 10% for both types of eCig liquid that was sufficient to exert changes without affecting cell behavior. At the conclusion of the exposure, total cell lysates and conditioned media were obtained.

### NF-kB and TNF-α Assay

Released Nuclear factor kappa B (NF-*k*B) and Tumor necrosis factor alpha (TNF-α) levels were assessed in culture media (n=8) using colorimetric high-throughput fast activated cell-based ELISA assays available from Active Motif (Carlsbad, CA). Specifically, cells were screened with antibodies specific to total and active phosphorylated proteins as outlined in the manufacturer’s instructions. *In vitro* experiments were repeated at least three times, each in triplicate.

### Western Blot

Western blot analysis (n=10) was performed as outlined by our lab previously (5). Briefly, cells were lysed in protein lysis buffer (RIPA; Fisher Scientific). Protein lysates (30 ug) were separated by electrophoresis through Mini-PROTEAN TGX Precast gels (Bio-Rad Laboratories, Hercules, CA, USA) and transferred to nitrocellulose membranes. Membranes were incubated overnight with antibodies against phospho (p)extracellular signal-regulated kinase (ERK), phospho (p) c-Jun N-terminal Kinase (JNK), MMP-9, MMP-13, or β-Actin (all from Cell Signaling, Danvers, MA, USA). For protein detection, membranes were then incubated with fluorescent secondary antibodies for an hour and washed ×3 with TBST the next day prior to imaging.

Membranes were developed on a Li-COR Odyssey CLx. Fluorescence densities were determined, and comparisons were made between treated and control groups.

### Real-Time Cell Invasion

Real-time cell invasion of cell lines (n=10) was assayed as done previously in our laboratory (5). Briefly, following the experimental treatments, invasion was quantified with a xCELLigence Real-Time Cell Analysis (RTCA) DP (dual purpose) instrument (ACEA Biosciences, Blue Springs, MO, USA) using 16-wellCIM-Plates (n=10; (ACEA Biosciences). Plate wells were coated with a 1:40 concentration of Matrigel (Fisher Scientific, Pittsburg, PA, USA) and OSCC cells were plated in the top chamber at a concentration of 20,000 cells in a total volume of 100 ul of 2% FBS medium. The bottom chamber wells were filled with 160 ul of RPMI containing 10% FBS and invasion readings were obtained every 15 min for 24 h.

### Statistical Analysis

Results were checked for normality, and data was shown as means ± SE. Differences in cell invasion, NF-*kB*, TNF-α, ERK, JNK, MMP-9 and MMP-13 were determined between control and treated cells. Mann-Whitney tests were used to compare the changes in the protein expression of NF-*k*B, TNF-α, ERK, JNK, MMP-9 and MMP-13 and the differences in invasion indexes. Significant differences between groups were noted at *P* <.05. Statistical analysis was performed with GraphPad Prism 8.0 software.

## Results

### Electronic Vapor Extract (EVE) and Inflammatory Markers in OSCC Cells

**Figure 1:**
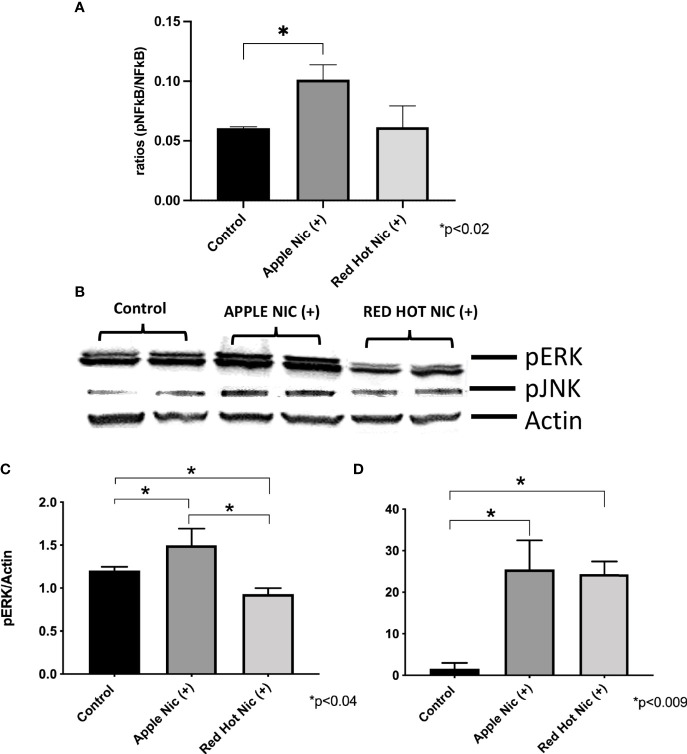
Expression of NF-*k*B, pERK, and pJNK in Ca9-22 cells exposed to EVE. ELISA was performed to determine NF-*k*B released into the cell medium. ERK and JNK were determine by western blot. Released of NF-*k*B in the media was increased only with Apple EVE and nicotine EVE in treated Ca9-22 cells as compared to media only treated controls **(A)**. A representative western blot results for ERK and JNK us shown in **(B)** ERK protein was activated in cells treated with Apple EVE and Nicotine and significantly decreased in cells exposed to Red Hot EVE with nicotine **(C)**. JNK protein was activated by both flavors in Ca9-22 cells when nicotine was present **(D)**. Data are shown with *p ≤ 0.05.

**Figure 2:**
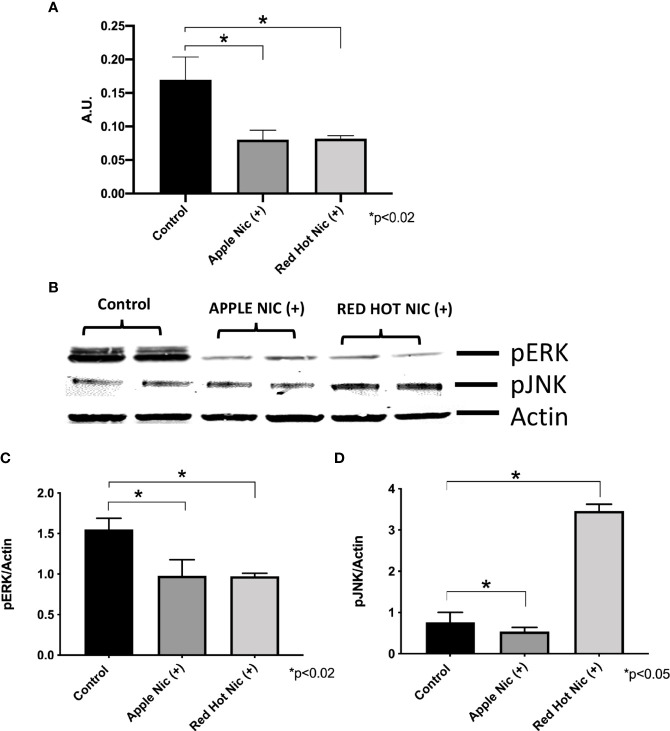
**|** Expression of NF-*k*B **(A)**, pERK **(B)**, and pJNK **(C)** in Cal27 cells exposed to EVE. ELISA was performed to determine NF-*k*B released into the cell medium. ERK and JNK were determine by western blot. Released NF-*k*B was decreased with both flavors and nicotine in these cells **(A)** as compared to media only treated controls cells. A representative western blot results for ERK and JNK is shown in **(B)** Activation of ERK and JUNK proteins protein was decreased after exposure to both flavoring with nicotine except for induction of pJNK by Red Hot **(C, D)**. Data are shown with *p ≤ 0.05.

Cigarette smoke environments are associated with pro-inflammatory signaling by OSCC cells (18). Expression of the mitogen activated protein kinases (MAPK) family (ERK, JNK) and NF-*k*B are activated and they are known to be involved in the regulation of cell proliferation and the induction of immune responses (19). We first investigated expression of NF-*k*B, ERK and JNK in Ca9-22 or Cal27 cells. A representative western blot for Ca9-22 ERK and JNK results is shown in **Figure 1A**. Apple and Red Hot EVE treatment alone did not show differences in NF-kB, ERK of JNK in Ca9-22 and Cal27cells as compared to controls (data not shown). When nicotine was added to treated Ca9-22 cells, Apple EVE increased NF-kB (1.5-fold; p<0.02) while no differences were observed with Red Hot EVE (**Figure 1A**). A representative western blot for Ca9-22 ERK and JNK results is shown in **Figure 1B**. Apple EVE and nicotine treatment increased ERK activation by Ca922 cells (1.3-fold; p<0.04) but there were no differences in ERK expression when cells were treated with Red Hot EVE (**Figure 1C**). Addition of nicotine increased JNK activation when cells were treated with both Apple (32.8-fold; p<0.09) and Red Hot EVE (40.2-Fold; p<0.009; **Figure 1D**). We next compared NF-kB, ERK and JNK in the Cal27 cells. NF-kB was decreased in Cal27 when treated with both Apple (2.1-fold p<0.02) and Red Hot EVE (2.1-Fold; p<0.02) when compared to controls (**Figure 2A**). A representative western blot for Cal27 ERK and JNK results is shown in **Figure 2A**. Activation of ERK was decreased by both Apple EVE (1.8-Fold; p>0.02) and Red Hot (1.7; p<0.02) in Cal27 cells (**Figure 2C**). Activation of JNK was decreased (1.4-fold; p<0.05) in Cal27 cells treated with Apple EVE and nicotine while JNK activation was increased (6.4-fold; p<0.05) when cells were treated with Red Hot EVE and nicotine (**Figure 2D**).

### Electronic Vapor Extract (EVE), Tumor Necrosis Factor Alpha (TNF-α) and Cell Invasion

**Figure 3:**
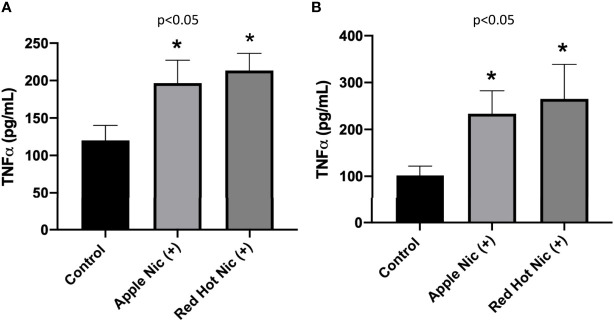
Expression of TNF-α in OSCCs exposed to EVE. ELISA was performed to determine TNF-α released into the cell medium. Released TNF-α levels were increased in conditioned media from Ca9-22 **(A)** and Cal27 **(B)** exposed to both EVE flavors plus nicotine. Data are shown with *p ≤ 0.05.

We next investigated TNF-α expression by OSCCs cells when treated with EVE. TNF-α plays a role in cellular responses to infection and during immune responses. More recently, a role has been established for TNF-α in the regulation of OSCC invasion and metastasis (20). TNF-α secretion was increased by Ca9-22 cells when exposed to either Apple (1.3-fold; p<0.05) or Red Hot (1.5-fold; p<0.05) EVE in the presence of nicotine (**Figure 3A**). Similarly, TNF-α was increased by Apple (2.2-Fold; p<0.05) and Red Hot (2.6-fold; p<0.05) EVE in Cal27 cells (**Figure 3B**).

**Figure 4:**
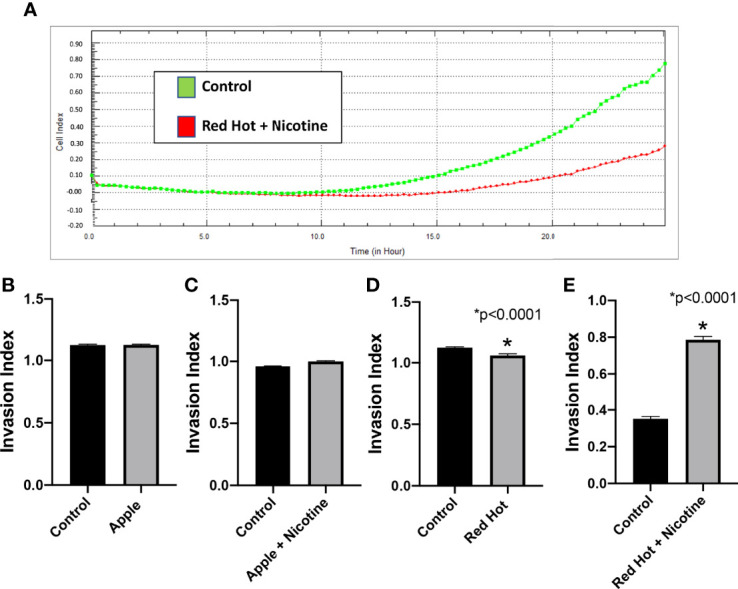
Ca9-22 invasion with Green Apple or Red Hot EVE. A representative diagram of readout cells over time invasion is shown in **(A)**. At 24 hours of culture, Green Apple EVE did not affect Ca9-22 invasion with or without nicotine **(B, C)**. Treatment with Red Hot EVE decreased Ca9-22 invasion **(D)** and Red Hot with nicotine enhanced Ca9-22 invasion **(E)** when compared to c untreated controls. Data are shown with *p ≤ 0.05.

**Figure 5:**
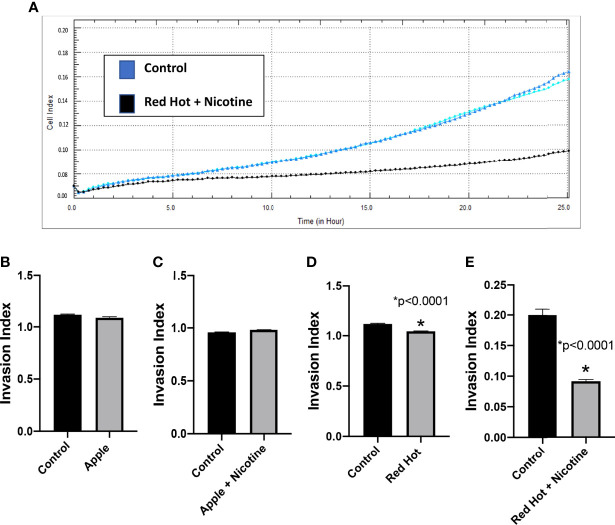
Cal27 invasion with Green Apple or Red Hot EVE. A representative diagram of readout cells over time invasion is shown in **(A)**. Green Apple EVE did not affect Cal27 invasion in cells with or without nicotine **(B, C)**. Red Hot EVE reduced Cal27 invasion in cells with and without nicotine **(D, E)** as compared to untreated controls. Data are shown with *p ≤ 0.05.

To further characterized EVE effects on OSCC, we investigated its effect on cell invasion. OSCC is characterized by high incidence of local invasion and metastasis that leads to poor prognosis (21). This invasiveness is known to be regulated in part by the presence of inflammatory markers (22). Our lab previously showed that eCig liquid flavoring and nicotine differentially regulate OSCC cell invasion (23). We therefore investigated OSCC cell invasion during EVE treatment. A representative graph of invasion results for Ca9-22 and Cal27 (Control vs Red Hot + Nicotine) is shown in **Figure 4A** and **Figure 5A**. No differences on cell invasion were observed when Ca9-22 cells were treated with Apple EVE in the presence or absence of nicotine (**Figures 4B, C**). In Ca9-22 cells, Red Hot EVE decreased cell invasion (1.44-fold; p<0.0001; **Figure 4D**). This decrease was reversed (2.0-fold decreased; p<0.0001) when nicotine was added to the Red Hot EVE treatment (**Figure 4E**). In Cal27 cells, Apple EVE treatment did not change cell invasion as compared to control (**Figures 5B, C**). Interestingly, Red Hot EVE treatment decreased Cal27 OSCC cell invasion (1.2-fold; p<0.0001) and this decrease was potentiated (2.2-fold; p<0.0001) when nicotine was added (**Figures 5D, E**).

**Figure 6:**
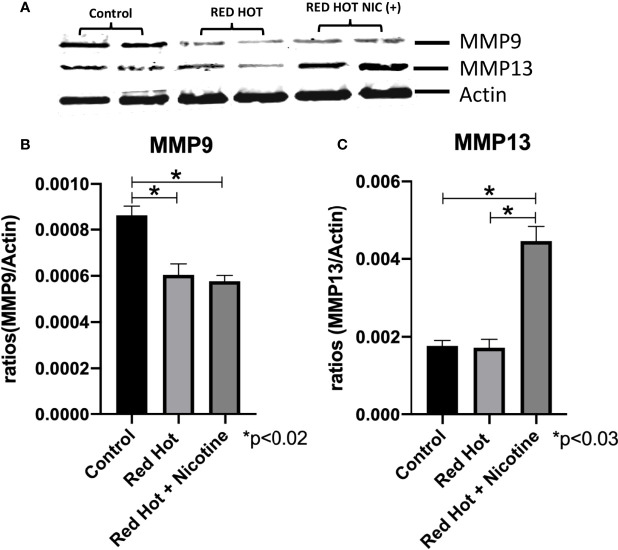
MMP-9 and MMP-13 during Red Hot EVE treatment of Ca9-22 cells. A representative Ca9-22 MMP9 and 13 western blot picture is shown in **(A)** Western blot analysis showed decreased MMP-9 when Ca9-22 cells were treated with Red Hot EVE in the presence or absence of nicotine **(B)**. MMP-13 was increased in cells exposed to Red Hot EVE with nicotine **(C)** when compared to untreated control cells. Data are shown with *p ≤ 0.05.

**Figure 7:**
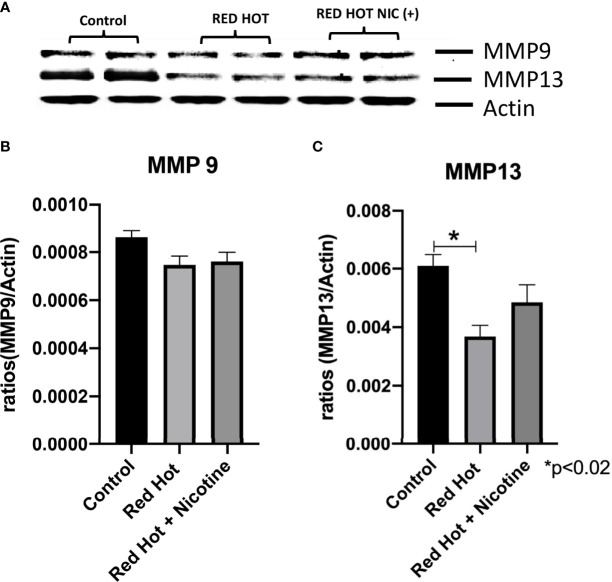
MMP-9 and MMP-13 during Red Hot EVE treatment of Cal27 cells. A representative Cal27 MMP9 and 13 western blot picture is shown in **(A)** Western blot analysis showed that MMP-9 did not change when cells were treated with Red Hot EVE with or without nicotine **(B)** when compared to untreated control cells. MMP-13 was decreased with Red Hot EVE treatment alone **(C)**. Data are shown with *p ≤ 0.05.

Matrix metalloproteinases (MMPs) are proteases that have vital roles in cell growth, invasion, and angiogenesis (24). MMP-9 is a protease which functions in many biological processes and has been widely found to be relate to the pathology of cancerous growth (25). MMP-13 is an additional protease that tends to be expressed during pathological situations and is particularly elevated by squamous cell carcinomas (24). We therefore investigated the levels of MMP-9 and MMP-13 and their correlation with OSCC cell invasion. As invasion differences were observed only with Red Hot EVE treatment, levels of these MMPs were determined only during Red Hot treatment. A characteristic Ca9-22 MMP 9 and 13 western blot results are shown in **Figure 6A**. Ca9-22 cells showed decreased MMP-9 expression in the absence (1.4-fold; p<0.002) or presence of nicotine (1.5-fold; p<0.002) on during EVE treatment (**Figure 6B**). In contrast, EVE treatment alone did not affect MMP-13 protein (**Figure 6C**), but the addition of nicotine led to significantly increased (2.9-fold; p<0.03) MMP-13 production (**Figure 6C**). A characteristic Cal27 MMP 9 and 13 western blot results are shown in **Figure 7A**. Cal27 cells exposed to Red Hot EVE did not alter MMP-9 protein regardless of nicotine (**Figure 7B**). MMP-13 was decreased (1.7-fold; p<0.02) when Cal27 cells were treated with Red Hot EVE, but this expression was reversed when nicotine was added to EVE treatment (**Figure 7C**).

## Discussion

eCigs vaporize and deliver liquid flavorings in the presence or absence of nicotine **(** 26 **).** This new avenue of tobacco has gained significant popularity in recent years, and it has been presented as less harmful as compared to traditional smoking. Knowledge about the toxicity of the e-liquid electronic devices is still scarce; in fact, the World Health Organization has stated eCig vaping should not *be* recommended until its true toxicity profile have been properly established (Food and Drug Administration, HHS, 2016). Previous studies in our laboratory showed diverse inflammatory and invasive effects employed by OSCCs when treated with eCig liquid and nicotine (23). This research suggested a flavor and nicotine dependent regulation of OSCC biology. To better understand eCig vapor effects, we exposed OSCCs with culture medium treated with the vapor generated by eCig delivery devices and termed the vapor as eCig extract (EVE). We observed that the inflammatory regulator NF-*k*B was increased by OSCC gingiva cells (Ca9-22) when treated with Apple EVE supplemented with nicotine. NF-*k*B signaling pathways are central coordinators of innate and adaptive immune responses and it orchestrates the communication between cancer cells and inflammatory cells (27). In fact, studies have correlated NF-*k*B and inflammation intensity with oral carcinogenesis (27). Together, these results suggest that Apple flavor induces the production of NF-*k*B, which may be involved in the production of immune responses by OSCC cells when nicotine is added to EVE. These results support the already established data showing that nicotine promotes oral carcinogenesis (28). ERK and JNK are members of the of the MAP kinase pathways that regulate cellular responses. The ERK pathway is usually associated with cell proliferation and survival, while the JNK pathway is usually associated with cellular stress. More recently, a role for the activation of these MAP kinase members has been demonstrated in enhancing proliferation and migration of OSCCs (29). We observed increases in the expression of both ERK and JNK when Ca9-22 OSCCs were treated with Apple EVE. These results suggest that activation of these pathways could be involved in OSCC progression and survival in the presence of EVE and nicotine. Like NF-*k*B, TNF-α also plays a vital role in the regulation of the inflammatory process encountered during tumor development, but studies have shown that NF-*k*B signaling causes TNF-α mediated invasion and metastasis of OSCCs (20, 30). We observed increased TNF-α in Ca9-22 cells in the presence of nicotine when treated with Apple EVE. This discovery correlates with the increased expression of NF-*k*B observed in Apple EVE treatment. Due to the fact that this correlation could be involved in an immune response or cell invasion, we decided to determine real time invasion in these cells after treatment. Our results did not show any invasive differences in these cells after treatment. These outcomes suggest that NF-*k*B and TNF-α activation could be more likely functional during an immune response rather than invasion when OSCCs were treated with Apple EVE and nicotine. To confirm this possibility, we determined the expression of two important metalloproteinases, (MMP) 9 and 13. MMPs are enzymes involved in the degradation of the extracellular matrix that increase the invasive properties of cells (31). MMP-9 and MMP-13 are 2 specific MMPs that are regulated in OSCCs (32). We did not observe upregulation of either of the MMPs in the Ca9-22 cells treated with Apple EVE, confirming the lack of cell invasion with this treatment.

We discovered upregulation of TNF-α but not NF-*k*B when Ca9-22 gingiva OSCCs were treated with the Red Hot and nicotine. It is already known that TNF-α is upregulated in OSCCs and perhaps our results could be clarified as a means of correlating upregulation of TNF-α with cell invasion (7). This idea may be validated by the fact that our results showed increased invasion in Ca9-22 cells when treated with Red Hot EVE and nicotine. Furthermore, increased invasion correlates well with increased MMP-13 in these cells when treated with Red Hot EVE and nicotine. Together these results suggest that increased invasion observed by gingiva OSCCs could be related to increased TNF-α and MMP-13 when cells are treated with Red Hot EVE and nicotine. Furthermore, ERK did not change in these cells while JNK activation was increased. This could be explained by the fact that ERK is associated with cell proliferation and perhaps at this point these cells are more involved in cell invasion. In terms of JNK, there are several reports that link activation of JNK to increased invasiveness of oral cells and raises the idea that perhaps JNK is involved in increased invasion observed by these cells (33–36).

The tongue OSCCs cells (Cal27) did not result in increased NF-kB, ERK, cell invasion, or MMP regardless of treatment type. However, we did observe increased production of TNF-α and increased activation of JNK with both treatments in these cells. As previously mentioned, a common feature of OSCC is increased TNF-α but its role in Cal27 cells seems to be different than tits function in treated Ca9-22 cells. The fact that ERK is decreased and JNK is increased in these cells, suggests that perhaps these cells are experiencing stress and no longer proliferative and, in such cases, TNF-α is potentially involved in stress signaling by the cell.

In summary, our work demonstrates that eCig vapor can induce inflammation and invasion of OSCCs in a cell type and flavor dependent manner. This study provides an important step in dissecting eCig vapor mediated mechanisms of cancerous invasion and may be helpful in determining key molecular avenues of invasion employed by OSCCs.

## Conclusion

The results summarized in this investigation could provide an important initial step in understanding electronic cigarette use and oral cancer and could lead to new avenues of study with possible therapeutic utility.

## Data Availability Statement

The original contributions presented in the study are included in the article/supplementary material. Further inquiries can be directed to the corresponding author.

## Author Contributions

JA and PR designed experiments and supervised data analysis; HR, CT, AR, EC, ER, LG, and SJ performed the experiments and edited manuscript; JA and PR planned and supervised the project and wrote the manuscript. LFW, DW, PR and JA edited and organized manuscript. All authors contributed to the article and approved the submitted version.

## Funding

This work was supported by a grant from the National Institutes of Health (1R15HL152257; PR and JA) and BYU Mentoring Environment Grants (PR and JA).

## Conflict of Interest

The authors declare that the research was conducted in the absence of any commercial or financial relationships that could be construed as a potential conflict of interest.

## Publisher’s Note

All claims expressed in this article are solely those of the authors and do not necessarily represent those of their affiliated organizations, or those of the publisher, the editors and the reviewers. Any product that may be evaluated in this article, or claim that may be made by its manufacturer, is not guaranteed or endorsed by the publisher.

## Abbreviations

OSCC, oral squamous cell carcinoma; Ca9-22, human oral squamous carcinoma cells; Cal27, human tongue squamous carcinoma cells; eCig, electronic cigarette; EVE, electronic cigarette vapor; SHS, second hand smoke; Red Hot, Red Hot e-cigarette flavoring; Apple; Reds Apple Juice e-cigarette flavoring; TNF-α, Tumor necrosis factor alpha; NF-*k*B, nuclear factor kappa B; ERK, extracellular signal-regulated kinase; JNK, c-Jun N-terminal Kinase; MMP, matrix metalloproteinases.
